# Global, segmental and layer specific analysis of myocardial involvement in Duchenne muscular dystrophy by cardiovascular magnetic resonance native T1 mapping

**DOI:** 10.1186/s12968-021-00802-8

**Published:** 2021-10-14

**Authors:** Ke Xu, Hua-yan Xu, Rong Xu, Lin-jun Xie, Zhi-gang Yang, Li Yu, Bin Zhou, Hang Fu, Hui Liu, Xiao-tang Cai, Ying-kun Guo

**Affiliations:** 1 Department of Radiology, Key Laboratory of Obstetric and Gynecologic and Pediatric Diseases and Birth Defects of Ministry of Education, West China Second University Hospital, Sichuan University, 20# Section 3 South Renmin Road, Chengdu, 610041 China; 2 Department of Radiology, West China Hospital, Sichuan University, Chengdu, China; 3 Department of Pediatric Cardiology, West China Second University Hospital, Sichuan University, Chengdu, China; 4 Laboratory of Molecular Translational Medicine, Center for Translational Medicine, West China Second University Hospital, Sichuan University, Chengdu, China; 5 Department of Radiology, Second Affiliated Hospital of Nanchang University, Nanchang, China; 6 Department of Pediatrics Neurology, West China Second University Hospital, Sichuan University, 20# Section 3 South Renmin Road, Chengdu, 610041 China

**Keywords:** Duchenne muscular dystrophy, Cardiovascular magnetic resonance, Native T1 mapping, Cardiomyopathy

## Abstract

**Background:**

Progressive cardiomyopathy accounts for almost all mortality among Duchenne muscular dystrophy (DMD) patients.‍ Thus, our aim was to comprehensively characterize myocardial involvement by investigating the heterogeneity of native T1 mapping in DMD patients using global and regional (including segmental and layer-specific) analysis across a large cohort.

**Methods:**

We prospectively enrolled 99 DMD patients (8.8 ± 2.5 years) and 25 matched male healthy controls (9.5 ± 2.5 years). All subjects underwent cardiovascular magnetic resonance (CMR) with cine, T1 mapping and late gadolinium enhancement (LGE) sequences. Native T1 values based on the global and regional myocardium were measured, and LGE was defined.

**Results:**

LGE was present in 49 (49%) DMD patients. Global native T1 values were significantly longer in LGE-positive (LGE +) patients than in healthy controls, both in basal slices (1304 ± 55 vs. 1246 ± 27 ms, p < 0.001) and in mid-level slices (1305 ± 57 vs. 1245 ± 37 ms, p < 0.001). No significant difference in global native T1 was found between healthy controls and LGE-negative (LGE−) patients. In segmental analysis, LGE + patients had significantly increased native T1 in all analyzed segments compared to the healthy control group. Meanwhile, the comparison between LGE− patients and healthy controls showed significantly elevated values only in the basal anterolateral segment (1273 ± 62 vs. 1234 ± 40 ms, p = 0.034). Interestingly, the epicardial layer had a significantly higher native T1 in LGE− patients than in healthy controls (p < 0.05), whereas no such pattern was noticed in the global myocardium. Epicardial layer native T1 resulted in the highest diagnostic performance for distinguishing between healthy controls and DMD patients in receiver operating curve analyses (area under the curve [AUC] 0.84 for basal level and 0.85 for middle level) when compared to global native T1 and endocardial layer native T1.

**Conclusions:**

Myocardial regional native T1, particularly epicardial native T1, seems to have potential as a novel robust marker of very early cardiac involvement in DMD patients.

*Trial registration:* Chinese Clinical Trial Registry (http://www.chictr.org.cn/index.aspx) ChiCTR1800018340, 09/12/2018, Retrospectively registered.

## Background

Duchenne muscular dystrophy (DMD), an X-linked muscle degenerative disorder caused by deficient or defective synthesis of dystrophin protein, is the most common inherited muscular dystrophy and affects both skeletal muscle and myocardial muscle. Due to improved ventilatory assistance and supported treatments, cardiomyopathy has become the main cause of death in DMD patients [1, 2]. Histopathological studies have revealed that subepicardial muscle degeneration, fibrosis, and fatty infiltration are the most common forms of myocardial injury in DMD‍ [3, 4]. In order to postpone the onset of cardiac remodeling and subsequent dysfunction, it is important to detect myocardial changes early, when they are still at a subclinical level [5]. Cardiovascular magnetic resonance (CMR) offers an accurate and highly reproducible technique for assessing left ventricular (LV) function together with the ability to detect focal fibrosis based on late gadolinium enhancement (LGE) imaging, which plays an increasingly vital role in the diagnosis and clinical care of boys with DMD-associated cardiomyopathies [6] and is thus recommended as the preferred noninvasive imaging modality for patients with DMD [7]. However, LGE imaging has several underlying limitations in its ability to display subtle or diffuse myocardial fibrotic changes due to the lack of regional differences in signal intensity [8]. Native T1 mapping, a novel CMR parametric mapping technique, has provided a potential tool for quantifying tissue alterations without the administration of contrast medium and assessing early, subclinical cardiac involvement in focal and diffuse myocardial fibrosis [9–11]. Increased myocardial native T1 in patients with DMD has been described in previous studies [12–15]. However, those studies used older DMD patients, and were focused only on the global myocardium of the mid-ventricle rather than a systematic quantification of layer-specific native T1 ranging from the epicardial to the endocardial layer. Thus, the aim of our study was to comprehensively characterize myocardial involvement by investigating the heterogeneity of native T1 mapping in a large young DMD population using global and regional (including segmental and layer-specific) analysis and to further assess the feasibility and specificity of native T1 mapping in the early detection of myocardial involvement in DMD.

## Materials and methods

### Study population

From July 2018 to January 2020, 99 DMD patients were prospectively recruited for this Institutional Review Board (IRB)-approved study. DMD diagnosis was confirmed by genetic testing and/or skeletal muscle pathology in all patients. The exclusion criteria were claustrophobia; severe arrhythmia; contraindications for the use of contrast media, such as severe renal insufficiency; and inability to cooperate during CMR. Twenty-five age-matched healthy males undergoing CMR with gadolinium for a separate clinical indication with normal results and no evidence of cardiovascular disease were included as a control group. Their indications for CMR were: chest pain (N = 9); syncope (N = 4); occasional atrial premature beats (N = 5); and poor image quality on prior echocardiogram (N = 7). Every subject or guardian provided written informed consent form before CMR examination.

### CMR imaging protocol

CMR scanning was conducted using a clinical 3 T CMR scanner (MAGNETOM Skyra, Siemens Healthineers, Erlangen, Germany) equipped with an 18-channel receiver coil. CMR protocols included cine, T1 mapping, and LGE sequences. In order to quantify cardiac structure and function, 8–12 continuous sections were acquired from the mitral valve level to the LV apex in the short-axis view with a balanced steady-state free precession pulse (bSSFP) sequence (echo time [TE] = 1.48 ms; repetition time [TR] = 3.42 ms; flip angle = 34°; slice thickness 8 mm; matrix = 126 × 224 pixels; field of view [FOV] = 300 × 241 mm^2^). Matched T1 mapping and LGE imaging sequences were performed at three standard short-axis levels (basal, middle, and apical) in the LV. Prior to contrast administration, native T1 mapping was performed using a modified Look-Locker inversion recovery (MOLLI) sequence with motion correction (MOCO) [16]. The scanning model of MOLLI sequence was 5(3)3 (TE = 1.11 ms; TR = 2.71 ms; flip angle = 35°; slice thickness = 6 mm; matrix = 139 × 192 pixels; FOV = 280 × 224 mm^2^). LGE images were obtained 5–8 min after intravenous injection of gadolinium (Gadovist, Bayer Healthcare, Berlin, Germany) at a dose of 0.15 mmol per kg body weight by using a single-shot phase-sensitive inversion recovery (PSIR) sequence (TE = 1.09 ms; TR = 2.55 ms; flip angle = 55°; slice thickness = 6 mm; matrix = 116 × 192 pixels; FOV = 340–360 × 340–360 mm^2^). All images were acquired during breath holding in end-expiration, and electrocardiographic gating was used.

### Image analysis

**Fig. 1:**
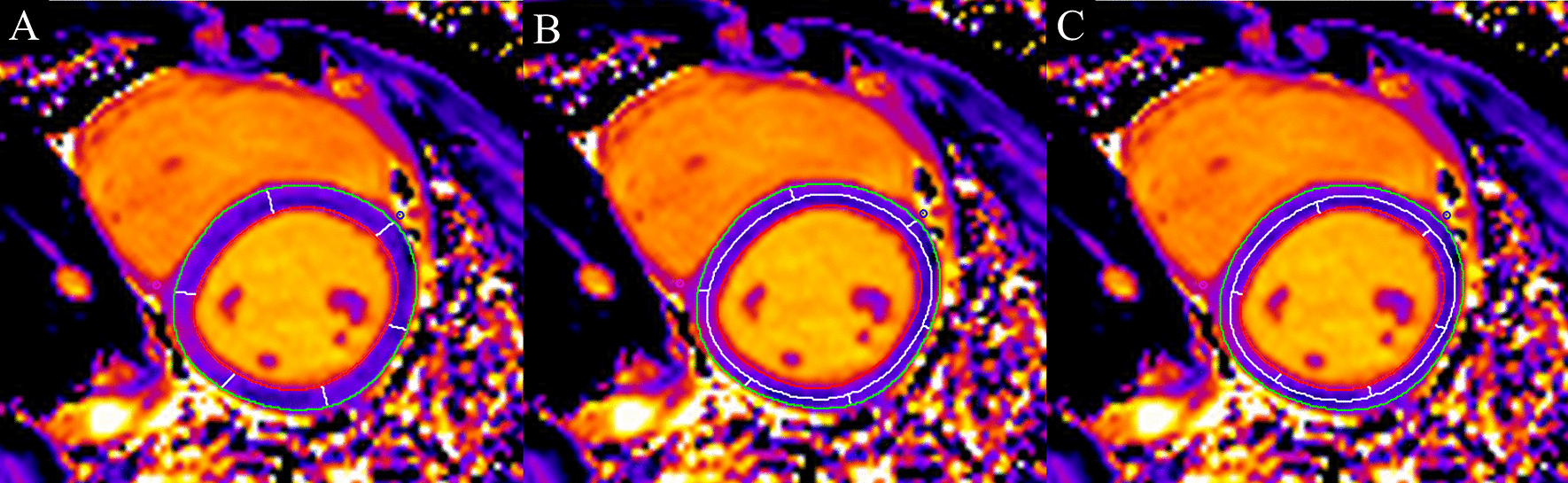
An example of outlining the regions of interest to obtain average native T1 values for the global myocardium (**A**), epicardial layer (**B**) and endocardial layer (**C**)

CMR analysis was performed offline by two experienced clinicians using commercially available software (cvi42; Circle Cardiovascular Imaging Inc., Calgary, Alberta, Canada). The LV function parameters, including LV ejection fraction (LVEF), end-diastolic volume index (EDVI), end-systolic volume index (ESVI), and LV mass index (LVMI), were derived by defining the contours of the endo- and epicardial borders on the short-axis cine images according to current guidelines [17]. LV dysfunction was defined as LVEF < 55%. Twelve segments (segments 1–12) of the LV (excluding the apical slice), adapted from the American Heart Association (AHA) 17-segment model [18], were analyzed through native T1 and LGE images. The apical slice was disregarded due to high artifact rate and the known error by partial volume. Using the cvi42 T1 characterization module, endocardial and epicardial borders were drawn on basal and middle ventricular T1 parametric maps for each patient. The anterior and posterior right ventricular (RV) insertion point was then defined to automatically divide the basal and middle ventricular slices into 6 segments and calculate segmental native T1 values. After removal of any segments affected by artifacts, the average T1 time for the global myocardium was calculated from the mean of the remaining segments. We apply an offset option of 50% by modifying the border contour towards the opposite border to generate native T1 of endo- and epicardial layers (see Fig. 1). We didn’t exclude areas of LGE as described recently [19]. As a test for intraobserver variability, one clinician performed all native T1 analyses a second time after a 1-month delay. The second radiologist, who was blinded to the results of the first observer, reanalyzed all the images to assess interobserver variability. The presence and pattern of LGE were visually assessed by a single expert reader according to the AHA 17-segment model. The number of affected segments was also indicated. LGE was deemed negative or positive in each of myocardial segments by visual rating and performed independent from the T1 analysis. A subject was considered to have cardiac involvement when LGE was present in at least one myocardial segment. If no enhancement was observed, then the subject was defined to have DMD without cardiac involvement [20]. Additionally, in DMD patients with cardiac involvement, the short-axis segments in the same slice position were further classified as positive segments or negative segments according to the presence/absence of LGE in each segment.

### Statistical analysis

Statistical analyses were performed using SPSS software (version 22.0 for Windows; Statistical Package for the Social Sciences, International Business Machines, Inc., Armonk, New York, USA) and MedCalc (version 19.7, MedCalc Software; Mariakerke, Belgium). Continuous variables are presented as the mean value ± standard deviation (SD). Categorical variables are expressed as the frequency and percentage. Normality was evaluated using the Kolmogorov–Smirnov test, and homogeneity of variance was assessed using Levene’s test. Student’s t-test was used to compare normally distributed characteristics between DMD and controls. The demographics of DMD patients and healthy controls were compared using a Wilcoxon rank-sum test. The CMR results of DMD patients and healthy controls were compared using an independent t-test. One-way ANOVA was used to compare the CMR results among DMD subgroups and the healthy control group, and the Bonferroni correction was used for to adjust the post hoc pairwise tests for multiple comparisons. A paired t-test was used to compare the native T1 values of the endocardial and epicardial layers. Receiver operating characteristic (ROC) analysis with area under the curve (AUC) was used to identify the best discriminating parameter in the myocardial layers to differentiate between DMD patients and controls. Interobserver and intraobserver variability were calculated using the intraclass correlation coefficient (ICC). All tests were 2-sided, and a p-value < 0.05 was considered statistically significant.

## Results

### Patient characteristics

The baseline characteristics of the patients are shown in Table 1. There were 99 DMD patients and 25 healthy controls. The mean age of the DMD patients was 8.8 ± 2.5 years (range 6–19 years), whereas the mean age of the controls was 9.5 ± 2.5 years (range 6–14 years). All subjects in both groups were males. We found three significant demographic differences between the two groups: DMD patients were shorter and had faster resting heart rates and lower body surface area (BSA) values than healthy controls (Table 1). Fourteen DMD subjects had an abnormal CMR-derived LVEF, defined as LVEF < 55%. DMD patients showed significantly lower LVEDVI, LVESVI and LVMI than healthy controls.

**Table 1:** Baseline characteristics and left ventricle characteristics

	Controls N = 25	All DMD patients N = 99	DMD LGE + N = 49	DMD LGE− N = 50
*Baseline characteristics*				
Age, years	9.5 ± 2.5	8.8 ± 2.5	9.2 ± 2.7	8.5 ± 2.4
Males (%)	25 (100%)	99 (100%)	49 (100%)	50 (100%)
Height, cm	135.7 ± 17.6	125.8 ± 12.5*	127.8 ± 12.3	124.2 ± 12.5*
Weight, kg	32.5 ± 10.5	28.5 ± 9.8	29.2 ± 8.9	27.9 ± 10.1
BMI, kg/m^2^	17.2 ± 2.5	17.5 ± 3.7	17.4 ± 3.2	17.6 ± 4.1
BSA, m^2^	1.8 ± 0.3	1.6 ± 0.2*	1.7 ± 0.2	1.6 ± 0.2*
Medications				
Corticosteroids	0	60 (60%)	34 (69%)	26 (52%)
ACEI	0	19 (19%)	10 (20%)	9 (18%)
β-blocker	0	20 (20%)	13 (27%)	7 (14%)
Diuretic	0	8 (8%)	6 (12%)	2 (4%)
*Left ventricle characteristics*				
Heart rate, bpm	80.9 ± 13.7	95.9 ± 14.8*	92.3 ± 18.6*	97.7 ± 16.6*
EF, %	62.0 ± 3.9	60.1 ± 8.0	57.3 ± 8.6*	62.7 ± 6.4^&^
EDVI, mL/m^2^	53.0 ± 10.7	43.5 ± 10.2*	46.9 ± 10.8	40.3 ± 8.7^*&^
ESVI, mL/m^2^	20.2 ± 4.9	17.3 ± 5.2*	19.9 ± 5.3	15.0 ± 3.8*^&^
LVMI, g/m^2^	30.1 ± 8.0	23.9 ± 6.0*	25.4 ± 6.1*	22.5 ± 5.6*

Values are presented as mean ± standard deviation or n (%)

*ACEI* angiotensin converting enzyme inhibitor, *BMI* body mass index, *BSA* body surface area, *EF* ejection fraction, *EDVI* end-diastolic volume index, *ESVI* end-systolic volume index, *LVMI* left ventricular mass index

*p < 0.05 vs. controls

^&^p < 0.05 vs. DMD LGE +

### LGE presence

**Fig. 2:**
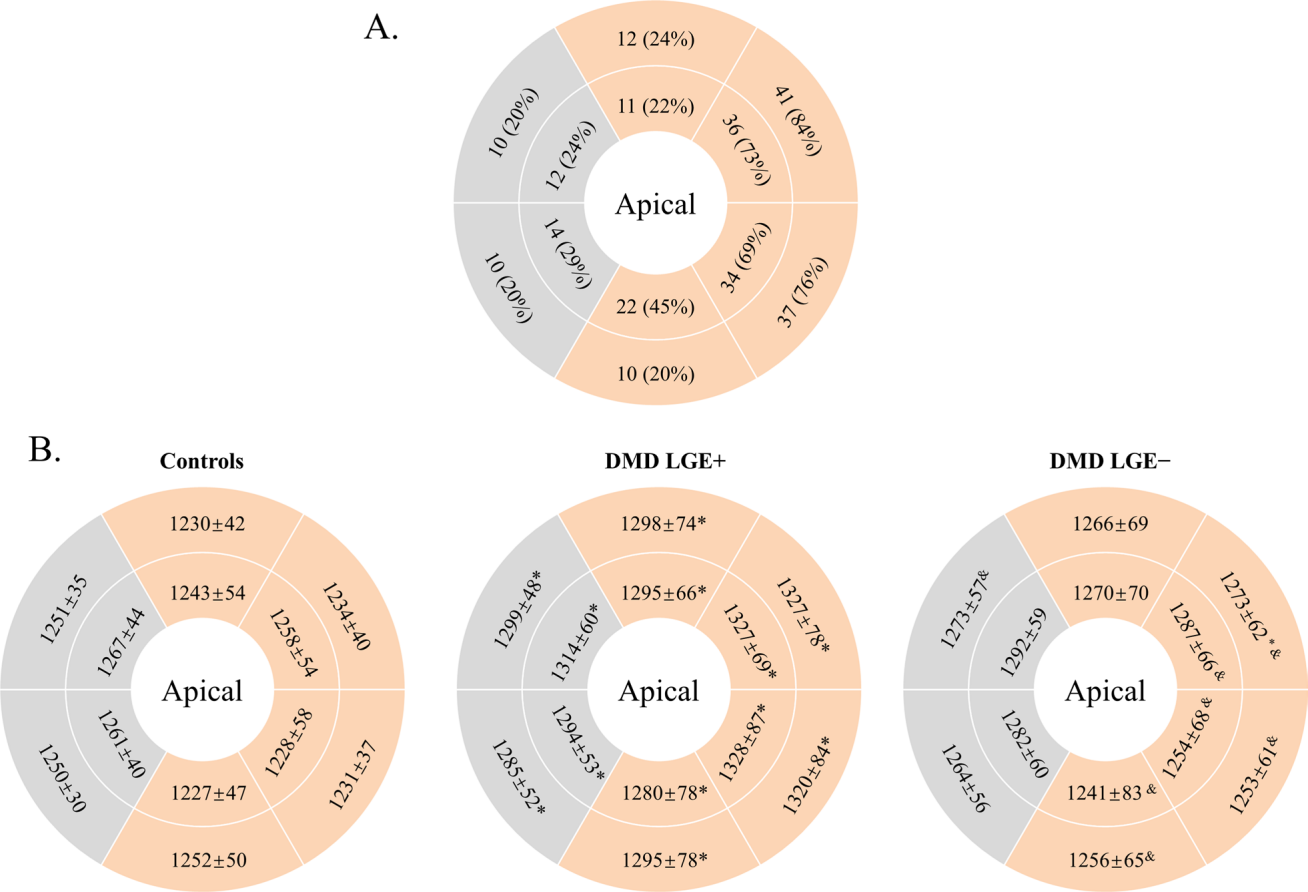
Schematic representation of bull’s-eye plots for the analyzed basal and middle short-axis slices, showing the following: **A** The presence and distribution of late gadolinium enhancement (LGE) -positive segments for all boys with Duchenne’s muscular dystrophy (DMD) with LGE +. The outer ring represents the basal segments, and the inner ring represents the midventricular segments. Free wall segments (orange) were more commonly affected by LGE than septal segments (gray). **B** Segmental native T1 values (means ± SD) in controls (left), DMD patients with LGE + (middle) and DMD patients with LGE− (right). Segments with abnormal values compared to healthy controls are marked with asterisks. Segments with abnormal values compared to LGE + patients are marked with the symbol &. (^*^p < 0.05 vs. controls; ^&^p < 0.05 vs. DMD with focal fibrosis.)

Among the 99 DMD patients, LGE was found to be present in 49 subjects (49%), defined as the LGE-positive (LGE +) group. The remaining 50 patients (51%) were assigned to the LGE-negative (LGE−) group. The baseline characteristics of LGE + patients were similar to those of LGE− patients, with the following exceptions: LGE + patients had a lower LVEF (57.3 ± 8.6% vs. 62.7 ± 6.4%, p = 0.010) and a significantly higher EDVI (p = 0.008) and ESVI (p < 0.001) than LGE− patients (Table 1). Figure 2A shows the presence and distribution of LGE + segments in all LGE + patients. Overall, LGE was mostly distributed in the subepicardial region, followed by the subendocardial region. In addition, LGE was more prevalent in the free wall segments than in the septal segments. At the basal and middle ventricle, the most frequently involved LV segments were the anterolateral [n = 77 (79%)] and inferolateral [n = 71 (72%)] segments. The less commonly involved segments were the anteroseptal [n = 22 (22%)] and inferoseptal segments [n = 24 (24%)].

### Global myocardial native T1 in different subcohorts of DMD patients

Global native T1 values were significantly higher in LGE + patients than in controls, both in the basal slices (1304 ± 55 vs. 1246 ± 27 ms, p < 0.001) and in the mid-level slices (1305 ± 57 vs. 1245 ± 37 ms, p < 0.001). A significant difference between LGE + and LGE− patients remained in the basal and mid-level slices (p = 0.002 and p = 0.021, respectively). Although no significant difference was found, the global native T1 values of LGE− patients still tended to be higher than those of controls in both the basal (1246 ± 28 vs. 1268 ± 54 ms, p = 0.349) and middle slices (1245 ± 35 vs. 1277 ± 48 ms, p = 0.051).

### Regional myocardial native T1 heterogeneity: layer-specific native T1

**Fig. 3:**
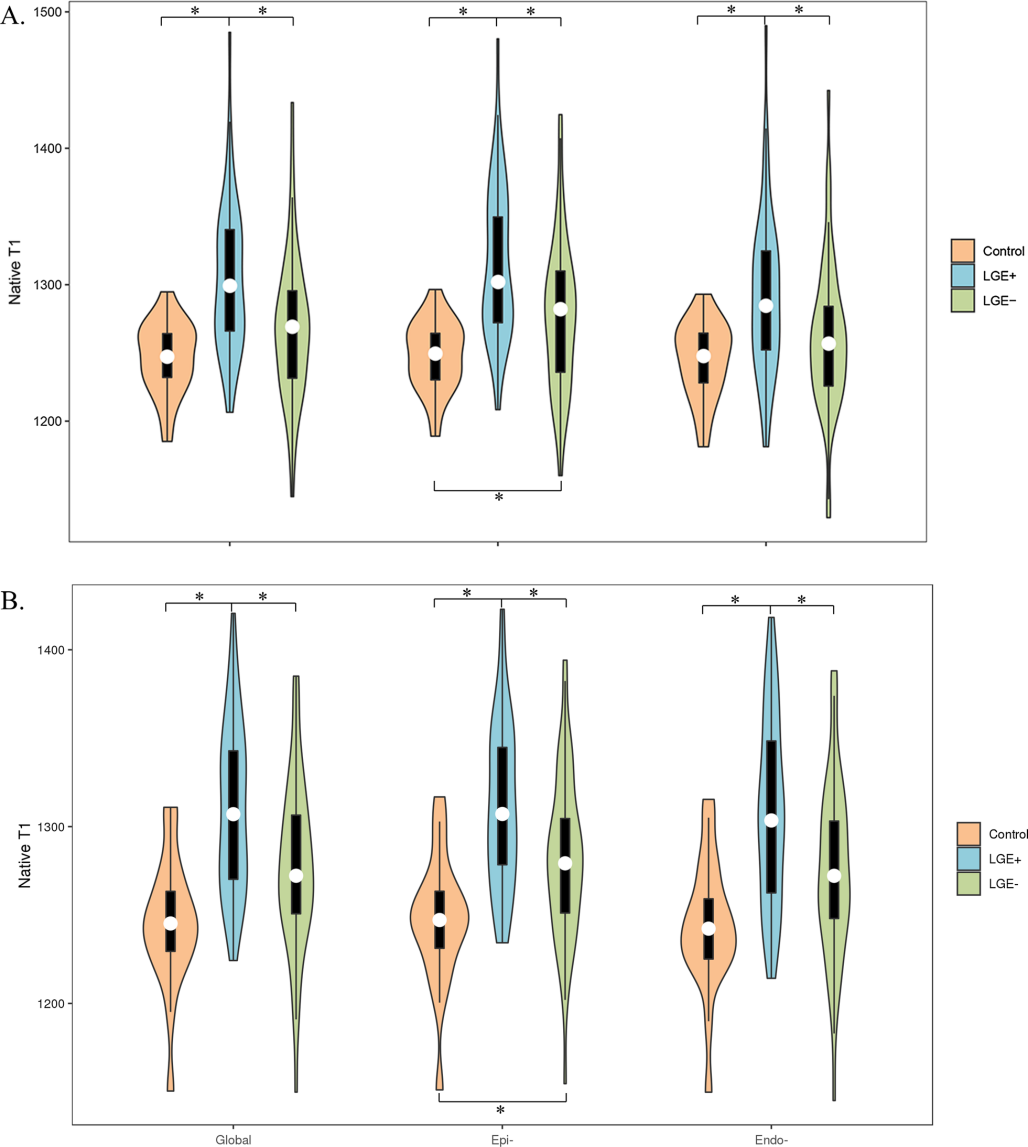
The violin plots of layer-specific native T1 values the DMD and healthy control groups at the basal (**A**) and mid-level (**B**) level

**Fig. 4:**
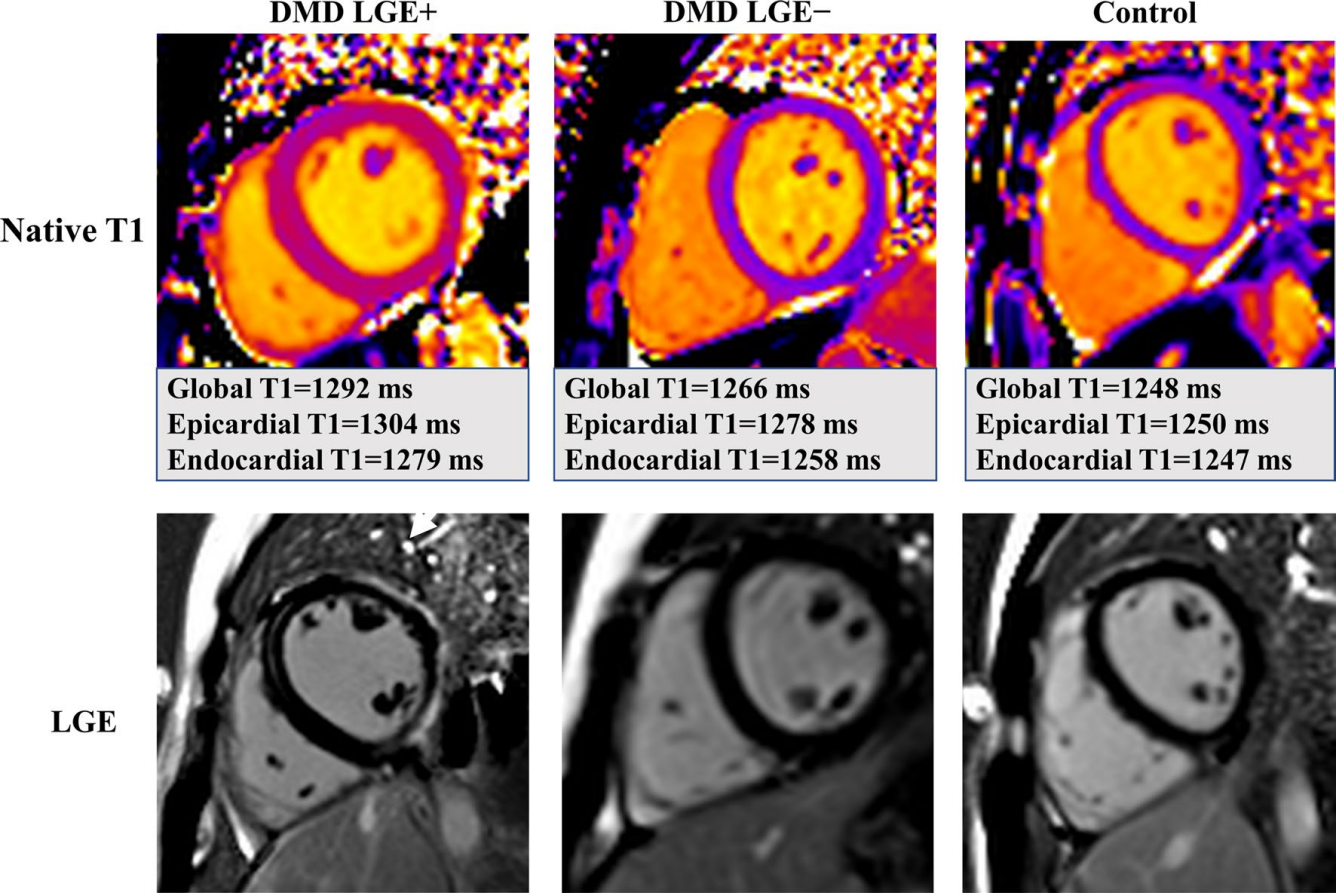
Example midventricular level short-axis native T1 maps (top) and corresponding late gadolinium enhancement (LGE) images (bottom). Example maps and images are shown for a DMD patient with cardiac involvement in the subepicardial layer of the free wall (left), for a DMD patient without cardiac involvement (middle), and for a healthy control (right)

**Fig. 5:**
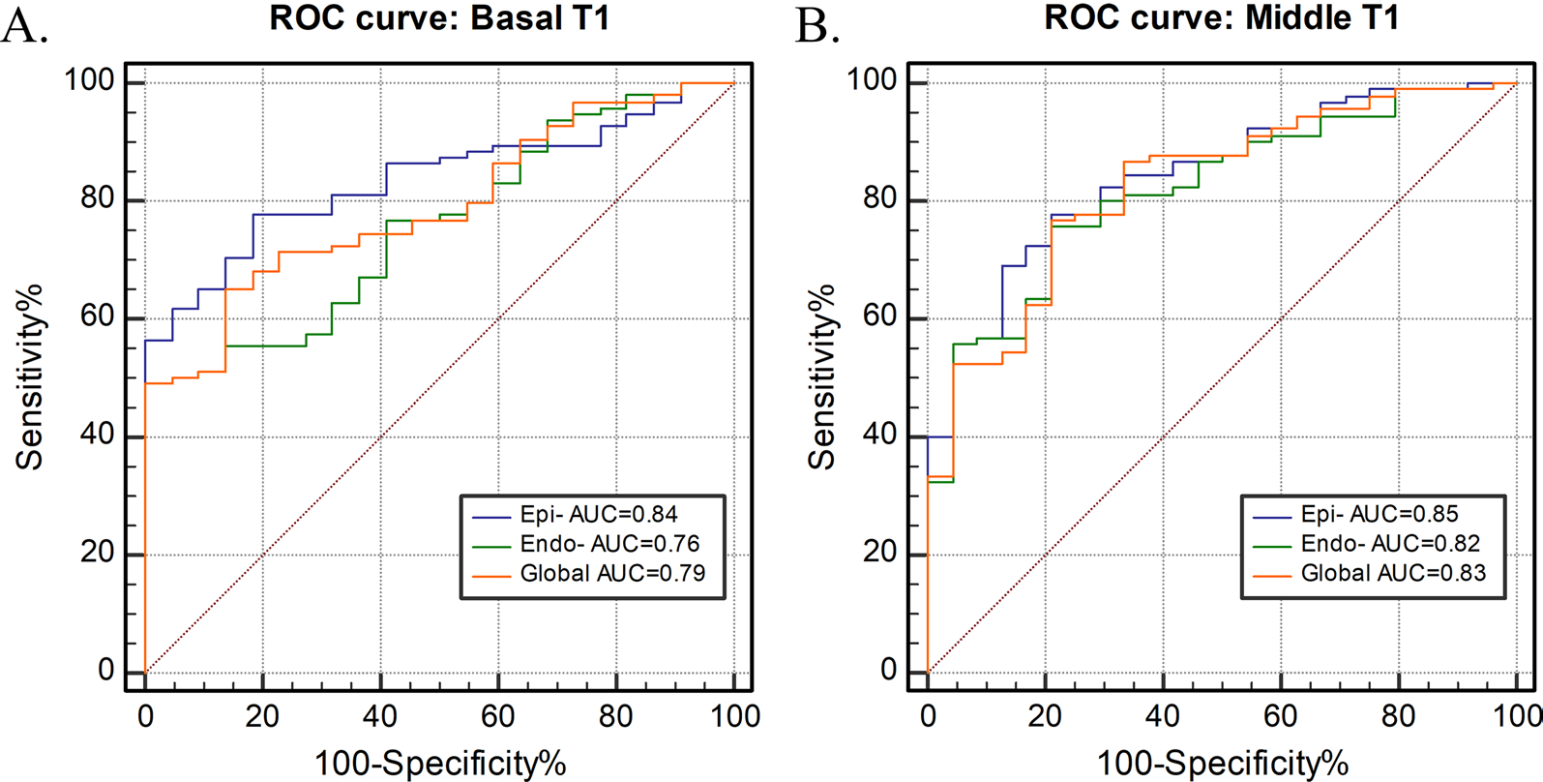
Receiver operating characteristic (ROC) curves for basal (**A**) and mid-level (**B**) T1-derived values for differentiating between healthy controls and DMD patients. *Endo-* endocardial layer, *Epi-* epicardial layer, *Global* global myocardium, *AUC* area under the curve

Native T1 values in different layers are depicted in Table 2. In the healthy control group, there was no difference in native T1 between the epicardial and endocardial layers. For LGE + patients, epicardial native T1 was significantly higher than endocardial native T1 at the basal level (1315 ± 56 vs. 1292 ± 59 ms, p < 0.001), but there was no significant difference at the mid-level (1307 ± 55 vs. 1303 ± 61 ms, p = 0.348). The same was observed in LGE– patients. Interestingly, the epicardial layer had a significantly higher native T1 in LGE− subjects than in healthy controls, which was not noticed in the global myocardium (Fig. 3). Figure 4 shows examples of native T1 maps with corresponding LGE images. The ROC curves plotted in Fig. 5 show that epicardial native T1 outperformed global and endocardial native T1 at distinguishing between healthy controls and DMD patients at both the basal level (AUC = 0.84) and the mid-level (AUC = 0.85).

**Table 2:** Comparison of global and layer-specific native T1 values

	Controls	DMD LGE + (N = 49)	DMD LGE− (N = 50)
*Basal*			
Global	1246 ± 27	1304 ± 55*	1268 ± 54^&^
Epi-	1248 ± 26	1315 ± 56*	1277 ± 54*^&^
Endo-	1245 ± 28	1292 ± 59*	1261 ± 56^&^
*Mid-level *			
Global	1245 ± 37	1305 ± 57*	1277 ± 48^&^
Epi-	1247 ± 36	1307 ± 55*	1282 ± 46*^&^
Endo-	1244 ± 37	1303 ± 61*	1276 ± 51^&^

*Endo*- endocardial layer, *Epi-* epicardial layer, *Global* global myocardium

*p < 0.05 vs. controls

^&^p < 0.05 vs. DMD LGE +

### Regional myocardial native T1 heterogeneity: segmental native T1

**Fig. 6:**
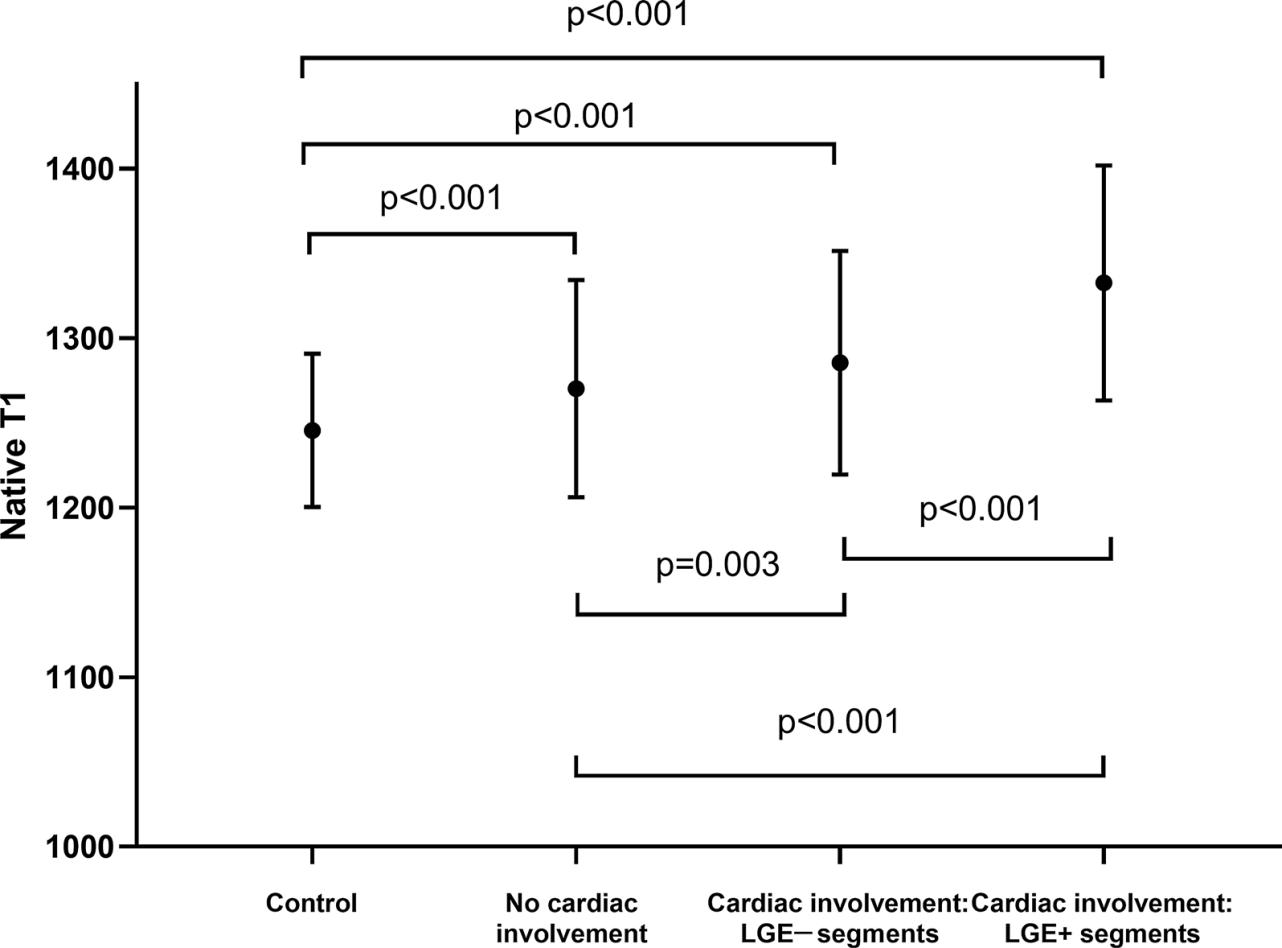
Even in the LGE- negative segments of DMD patients with cardiac involvement, the native T1 was significantly higher than those of patients without cardiac involvement and normal controls

27/1188 segments of 99 patients and 3/300 segments of 25 healthy control subjects had to be excluded due to artifacts and incorrect motion correction. The mean native T1 value of each segment is presented in Fig. 2B. LGE + patients had significantly increased native T1 in all analyzed segments in comparison with the healthy control population. The T1 elevation was distributed mainly in the lateral region. Note that even in septal segments with a low prevalence of LGE, the average native T1 of LGE + patients was elevated compared with that of controls. However, LGE− patients showed significantly higher native T1 than healthy controls only in the basal anterolateral segment (1273 ± 62 vs. 1234 ± 40 ms, p = 0.034). In addition, the segmental native T1 of LGE + patients was higher than that of LGE− patients for the inferior, inferolateral and anterolateral segments in both the basal and middle slices (p < 0.05). We also analyzed all AHA segments of each patient (a composite of the basal and middle ventricular slices) and further classified the segments in DMD patients with cardiac involvement as positive or negative segments according to the presence/absence of LGE. Our further analysis revealed substantially increased native T1 values not only in LGE-positive segments (1333 ± 69 ms) but also in LGE-negative segments (1286 ± 66 ms) of DMD patients with cardiac involvement compared to both DMD patients without cardiac involvement (1270 ± 64 ms) and controls (1246 ± 45 ms) (Fig. 6).

### Reproducibility analysis

The intra- and interobserver reliability of the measurement of the global native T1, epicardial native T1, and endocardial native T1 are shown in Table 3. Native T1 mapping showed good reproducibility (ICC = 0.968–0.996) in measuring myocardial involvement in DMD.

**Table 3:** Intra- and interobserver reliability of the measurement of global native T1, epicardial native T1, and endocardial native T1, described with ICCs and 95% CIs

Measurement	ICC (95% CI)	
	Intraobserver	Interobserver
Global native T1	0.996 (0.981–0.999)	0.991 (0.960–0.998)
Epicardial native T1	0.991 (0.973–0.997)	0.984 (0.946–0.995)
Endocardial native T1	0.968 (0.907–0.989)	0.981 (0.940–0.994)

*ICC* intraclass correlation coefficient, *CI* confidence interval

## Discussion

This study demonstrates the value of a comprehensive CMR-based quantification of native T1 in the diagnosis of DMD. Compared with global native T1 values, regional native T1 values provide a superior way to distinguish DMD patients from controls. To the best of our knowledge, this is the first study to investigate a method for quantitative evaluation of native T1 in specific layers of the myocardium. Our patients covered a wide age range. Interestingly, myocardial LGE-negative segments in DMD with cardiac involvement showed higher T1 values than those of either DMD without cardiac involvement or controls, indicating that a diffuse ongoing process in the myocardium can be observed in this genetic disease. In addition, the study provides further evidence to support the contention that fibrotic substitution in the myocardium of DMD patients usually has a heterogeneous distribution and predominantly proceeds from the epicardium to the endocardium [4, 21].

Despite advances in the diagnosis and management of DMD over the past 10 years, cardiomyopathy-related heart failure and arrhythmias are increasingly important sources of morbidity and mortality in DMD patients [22, 23]. This underlines the impact of an early diagnosis, as shown by Yilmaz et al. [24] using CMR. Nevertheless, due to young age and clinical presentation in the population, the decision is often difficult. Only 30% of boys with DMD have cardiac symptoms at diagnosis (far fewer than in other dilated cardiomyopathies). However, DMD cardiomyopathy carries higher mortality than other dilated cardiomyopathies [25]. Additional information about myocardial tissue changes could be helpful in guiding therapeutic decision making in certain circumstances. LGE imaging has served as the workhorse technique for tissue characterization; however, the inability to detect diffuse fibrosis with this technique makes it a restricted biomarker with regard to early detection and the efficacy of cardioprotective medications. Meanwhile, T1 mapping has been introduced and histologically validated to detect diffuse myocardial damage [26]. The present prospective analysis suggests that native T1 and extracellular volume (ECV) are robust indicators of myocardial disease associated with DMD [15, 27]. However, ECV is limited by the need for contrast administration, which may be considered invasive and makes it challenging for pediatric patients to endure. This study provides further evidence to support noncontrast examinations in pediatric DMD patients, particularly in conditions when the use of contrast might be contraindicated.

The native T1 values reported here are consistent with a recently published pediatric study by Maforo et al. using 3 T imaging [15]. However, our study provides the first comprehensive assessment of global and regional native T1 from basal to middle slices and from the epicardial layer to the endocardial layer. Our study found statistically significant differences in lateral but not septal native T1 between patients with and without focal fibrosis, a finding also demonstrated by Olivieri [14]. Soslow et al. [12] studied 31 DMD patients and found increased global native T1 relative to healthy controls not only among DMD patients in general but also in LGE-negative patients. One other group has also reported elevated global native T1 in LGE− patients compared with healthy subjects [13]. In contrast, LGE− patients in our study did not have increased global native T1 compared with healthy controls. It seems possible that this discrepancy is due to differences in the patient samples, particularly age and disease stage. For example, the population of the present study was younger than that of the study by Soslow et al. [12] (mean age 13.4 years) and had a higher LVEF (60.1% vs. 54.8%).

In light of the current knowledge, averaging native T1 over the entire myocardium may not be sensitive enough to detect regional abnormalities; accordingly, we conducted a layer-specific analysis as well. The results of this study demonstrated significant differences in the epicardial layer between DMD subjects without cardiac involvement and controls. In addition, native T1 of the epicardial layer provides the greatest distinction between DMD patients and healthy controls. We hypothesized that native T1 of the epicardial layer could provide a superior way to distinguish DMD patients from healthy controls. Hence, although global values seem easier to manage, postprocessing that facilitates the use of segmental or layer-specific evaluations is required in the future.

In postmortem hearts, it has already been demonstrated that myocardial damage starts in the subepicardial layer of the free wall with possible transmural extension in contiguous segments. This fibrotic change starts generally at the region behind the posterior and mitral valve apparatus and spreads downward progressively toward the apex [4, 24, 28]. The reasons why there was no significant difference between endocardial native T1 and epicardial native T1 at the middle level both in our LGE + and LGE− patients may be that fibrotic substitution in our LGE + patients only involved in basal slice in general and hasn’t progressed to the middle level due to their relatively young age. Since Silva et al. [29] initially reported the presence of LGE in DMD, many groups have demonstrated that LGE can reveal extracellular matrix (ECM) expansion consistent with fibrosis in the same location [30, 31]. Our results obtained in the present study by the state-of-the-art noninvasive technique of parametric mapping confirm those previous results and support the current belief that myocardial damage starts from the epicardial layer of the myocardium in DMD. Importantly, these changes are detectable even in DMD patients without apparent LGE and therefore provide an earlier indication of cardiac involvement.

### Limitations

Our study has several limitations. First, apical segments were not considered due to the high exclusion rate (60% had artifacts and/or partial volume effects), which prevents the AHA segmental analysis integrated. Additionally, the DMD group had significantly faster heart rates, which may influence the generalizability of our T1 mapping results [32]. However, guidelines suggest that the effects of heart rate on T1 mapping are minimized with the sequence parameters used in this study [33, 34]. Finally, we didn’t calculate ECV. Instead, we focused on the value of native T1 mapping rather than ECV quantification with contrast material. Native T1 measurement may be a replaceable method of ECV or LGE for detecting myocardial involvement, which is important in DMD patients because recent reports suggested that renal disease may be under-recognized in advanced-stage DMD patients [35].

## Conclusions

In DMD patients, native T1 measurement by CMR is a useful tool in assessing myocardial fibrosis as well as detecting myocardial abnormalities even in the absence of LGE. Myocardial regional native T1, particularly epicardial native T1, seems to have potential as a novel robust marker of very early cardiac involvement in DMD.

## Data Availability

The datasets used and/or analyzed during the current study are available from the corresponding author on reasonable request.

## Abbreviations

AHA: American Heart Association
AUC: Area under curve
BMI: Body mass index
BSA: Body surface area
bSSFP: Balanced steady-state free precession
CI: Confidence interval
CMR: Cardiovascular magnetic resonance
DMD: Duchenne muscular dystrophy
ECV: Extracellular volume fraction
ECM: Extracellular matrix
EDVI: End-diastolic volume index
ESVI: End-systolic volume index
ICC: Intraclass correlation coefficient
IRB: Institutional Review Board
LGE: Late gadolinium enhancement
LV: Left ventricle/left ventricular
LVEF: Left ventricular ejection fraction
LVMI: Left ventricular mass index
MOCO: Motion correction
MOLLI: Modified Look-Locker inversion recovery
PSIR: Phase-sensitive inversion recovery
ROC: Receiver operating characteristic
RV: Right ventricle/right ventricular
SD: Standard deviation
TE: Echo time
TR: Repetition time
